# Reference Values for 3D Spinal Posture Based on Videorasterstereographic Analyses of Healthy Adults

**DOI:** 10.3390/bioengineering9120809

**Published:** 2022-12-15

**Authors:** Janine Huthwelker, Jürgen Konradi, Claudia Wolf, Ruben Westphal, Irene Schmidtmann, Philipp Drees, Ulrich Betz

**Affiliations:** 1 Institute of Physical Therapy, Prevention and Rehabilitation, University Medical Center of the Johannes Gutenberg University Mainz, Langenbeckstraße 1, D-55131 Mainz, Germany; 2 Institute of Medical Biostatistics, Epidemiology and Informatics, University Medical Center of the Johannes Gutenberg University Mainz, Obere Zahlbacher Straße 69, D-55131 Mainz, Germany; 3 Department of Orthopedics and Trauma Surgery, University Medical Center of the Johannes Gutenberg University Mainz, Langenbeckstraße 1, D-55131 Mainz, Germany

**Keywords:** surface topography, rasterstereographic back shape analysis, normative data, healthy adults, posture analysis, spine

## Abstract

Visual examinations are commonly used to analyze spinal posture. Even though they are simple and fast, their interrater reliability is poor. Suitable alternatives should be objective, non-invasive, valid and reliable. Videorasterstereography (VRS) is a corresponding method that is increasingly becoming established. However, there is a lack of reference data based on adequate numbers of participants and structured subgroup analyses according to sex and age. We used VRS to capture the spinal posture of 201 healthy participants (aged 18–70 years) divided into three age cohorts. Three-dimensional reference data are presented for the global spine parameters and for every vertebral body individually (C7-L4) (here called the specific spine parameters). The vertebral column was found to be systematically asymmetric in the transverse and the coronal planes. Graphical presentations of the vertebral body posture revealed systematic differences between the subgroups; however, large standard deviations meant that these differences were not significant. In contrast, several global parameters (e.g., thoracic kyphosis and lumbar lordosis) indicated differences between the analyzed subgroups. The findings confirm the importance of presenting reference data not only according to sex but also according to age in order to map physiological posture changes over the life span. The question also arises as to whether therapeutic approximations to an almost symmetrical spine are biomechanically desirable.

## 1. Introduction

The spine connects the pelvis and the head with 24 vertebral bodies that can move against each other in three directions of movement. It stabilizes the torso and enables verticalization. The posture and movements of the spine are individually varied and highly characteristic of each person [1]. Visual inspection and posture analyses are important aspects of the basic examination of patients affected by spinal disorders [2]. Many musculoskeletal examiners have reported that visual estimations are one of their most commonly used assessment tools when analyzing spinal posture in clinical practice [3]. Although these visual assessments are simple and quick to perform, their results are relatively subjective, and their interrater reliability is statistically poor [3,4]. This becomes problematic when the results contribute to the clinical decision making process or are used in follow-up examinations to assess the progress and outcomes of the initiated therapies [5]. In order to address this problem, the collection of data regarding spinal posture should be objective and standardized using valid, reliable and reproducible measurement approaches. It is crucial for the assessments to be non-invasive for the patient and quick and easy to conduct in daily clinical routines. Videorasterstereography (VRS) seems to be a corresponding method that is increasingly becoming established in clinical practice [6,7,8].

The VRS system is based on a horizontal light line pattern projected onto the patient’s unclothed back and creates a virtual plaster cast of the individual back surface within only a few seconds [7]. In addition to information about the surface topographic curvature picture, the system is able to precisely estimate the position of every vertebral body (from C7 to L4) and the pelvis in a virtually constructed three-dimensional model of the human vertebral column [7,9,10,11,12]. VRS has evolved since its initial development in the 1980s and has been described in various publications [6,7,13]. The system has been proven to be valid and highly reliable compared to the clinical gold standard (X-ray imaging) [8,14,15,16,17].

In order to implement VRS for spinal posture analysis as a routine assessment in clinical practice, it is essential to have systematic reference data available for comparison with the potential pathological findings. Unfortunately, the current datasets are only conditionally able to fulfill these requirements, as they have several limitations.

Thus far, there are reference data for the global spine parameters of children [18], young adults [19,20,21] and young and middle-aged adults [22,23]. Either relatively heterogeneous study cohorts with very small numbers of participants have been analyzed without any further subgroup specifications [22,23], or subgroup-analyses have focused only on the potential differences between female and male participants in young, relatively homogenous study cohorts [19,20,21]. Possible changes in physiologic spinal posture according to sex and/or age over the adult life span have not yet been investigated. This knowledge, however, is essential for the consultation of reference data in clinical practice, in which not only young but also older patients are examined using VRS measurement devices.

In order to close this gap in our knowledge, the first aim of the current study was to provide practitioners and researchers with an additional set of VRS reference data that, firstly, included a preferably high number of healthy participants. Secondly, structured subgroup analyses were used depict possible physiologic changes in the spinal posture parameters according to sex and age over an adult life span of 18 to 70 years.

The second aim of this study was to provide the respective reference data for specific spine parameters: the isolated position of each vertebral body from C7 to L4 in all three dimensions of movement. These data are currently missing from the literature. As of up to a few years ago, only global spine parameters such as the thoracic kyphosis and lumbar lordosis angles were exportable from the DICAM 3 software. Meanwhile, the three-dimensional position of each vertebral body can be analyzed using an additional export interface.

In contrast to the work previously published by our own research group, describing a subgroup analysis of 100 asymptomatic females based on the dataset included here [24], this project involved a more differentiated analysis providing reference data for three different age cohorts (18–30 years, 31–50 years and 51–70 years) and for both sexes, respectively.

## 2. Materials and Methods

The data analyzed in this work were part of a prospective, explorative, cross-sectional and monocentric study assessing the three-dimensional spinal posture and movement behavior of healthy participants in the upright standing position and at four different walking speeds (2 km/h, 3 km/h, 4 km/h and 5 km/h). Ethical approval was obtained from the responsible ethics committee of the Rhineland-Palatinate Medical Association, and the study is registered with the World Health Organization (WHO) (INT: DRKS00010834). Based on a statistical sample size calculation, 201 healthy participants (sex ratio of 2/3 females to 1/3 males, aged 18–70 years) who gave their informed consent prior to participation were included in three different age cohorts (young (18–30 years), middle (31–50 years) and old (51–70 years)).

### 2.1. Participants

In order to participate, the volunteers had to be free of pain, and due to data capture requirements, their body mass index (BMI) had to be ≤30.0 kg/m^2^. All the participants had to demonstrate adequate gait stability (timed up-and-go test [25]), an age- and sex-accorded walking speed (two-minute walk test [26]) and spinal function (back performance scale [27]), as well as an appropriate joint mobility in order, theoretically, to be able to perform a physiological gait pattern [28]. Interested volunteers were excluded from participation in cases where they reported a history of surgery or fracture between the spinal segments of C7 and the pelvis. Further exclusion criteria were medical or therapeutic treatments due to spinal or pelvic girdle complaints (C7-pelvis) within the last 12 months or medical or therapeutic treatments due to musculoskeletal problems (musculoskeletal system except for C7-pelvis) within the last six months prior to the investigation.

### 2.2. Experimental Setup and Data Capture

In the study, “4D average” posture analyses were performed on all the participants using the DIERS Formetric III 4D measuring device (software versions DICAM v3.7.1.7 (DIERS International GmbH, Schlangenbad, Germany) for the data collection and DICAM v3.5.0Beta11 (DIERS International GmbH, Schlangenbad, Germany) for the data export), a VRS system based on the principle of triangulation [13]. A slide projector, used as the optical equivalent to an inverse camera, projects horizontal and parallel light lines onto the unclothed back of the participant, who is standing upright on a treadmill (height: ~18 cm) at a predefined distance from the measuring device (~2 m), with the eyes looking towards a standardized point ~2 m away and 20 cm below the individual’s body height (measured from the ground). Twelve series recordings of the transformed line pattern (due to back surface curvatures) were captured for a period of 6 s with an associated camera system. The three-dimensional scatter plot derived (consisting of up to 150,000 individual data points, depending on the body size) was used to create a virtual plaster cast of the surface of the participant’s back. The three-dimensional position of the underlying spine and the pelvis was estimated based on this information in combination with a clinically validated correlational model [11,12,13].

**Figure 1:**
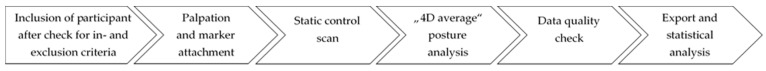
Schematic flow chart of the experimental process.

Even though it is technically not required for static VRS posture analyses, all the participants were marked with seven reflective markers prior to the data capture (on the spinal process of C7, the spinous processes between the medial parts of the spinae scapulae (~T3) and the thoracolumbar transitions (~T12), the left and right posterior superior iliac spine (PSIS) and on both acromia). This was necessary because the superior study protocol meant that the data for the dynamic gait analyses were also captured on the same measurement appointment. In order to best control for potential palpation or measurement bias, however, the same investigator (physical therapist) always performed the complete procedure themselves, including the entrance examinations (checking for inclusion and exclusion criteria), palpation, marker attachments and the VRS measurements, following a strict and standardized protocol. A static control scan was also performed to check for the correct placement of the markers. Where there were clinically inconclusive measurement results or any uncertainty on the part of the investigator, the placement of the markers was checked, palpated again, and corrected, if necessary, until the final marker position was defined. The measurements were repeated if the first graphical data output revealed clinically incomprehensible, inconsistent measuring artefacts or apparent software misinterpretations. For reasons of quality assurance, the investigator and an additional technician, who were both highly familiar with the software and the measuring device, further inspected all the pictures and the graphical data output visually after completion of the data collection phase for further abnormal spinal representations or other measuring artefacts and corrected them if necessary. In total, 46 specific and 14 global spine parameters were exported using the export interface of the DICAM v3.5.0Beta11 software. The Statistical Analysis System (SAS version 9.4) was used to combine all the exported files into one editable sheet of raw data. Figure 1 provides a schematic flow chart of the experimental process.

### 2.3. Data Analysis

The “4D average” measurement approach used meant that 12 individual values per participant were exported for every spine parameter. Several clinically inconclusive extreme values and, for one participant, isolated missing data points were identified in a preliminary visual data review. The Statistical Package of the Social Sciences (IBM SPSS Statistics for Windows, Version 23.0. Armonk, NY, USA: IBM Corp.) was used to systematically identify these values for every analyzed parameter, and all the extreme outliers revealed by the stem-and-leaf plot were removed from the raw dataset. The missing values were treated as extreme outliers, and the respective cells were removed from the raw dataset as well. The remaining values for every parameter were aggregated to finally create one mean value for every participant and for every parameter of interest.

Descriptive statistics were used to describe the reference values for all the specific (C7–L4 and the pelvis) and global spine parameters according to the mean of means (MoM) and the standard deviation (SD) in all three dimensions for the entire group, for all the female and all the male participants, and for the female and male participants within the three different age cohorts, respectively. An explorative two-way analysis of variance (two-way ANOVA) was used to check for possible differences between the groups according to sex, age cohort or a combination of both (level of significance *p* < 0.05). Possible deviations from the symmetrical zero positions of the different spine parameters were checked by one-sample Wilcoxon signed rank tests (level of significance *p* < 0.05). Graphical figures were created using Microsoft Excel (Microsoft Corporation, Version 2016. Redmond, WA, USA).

The authors do not include a detailed definition or description of the analyzed global and specific spine parameters. Instead, the reader is referred to the respective previous publications [19,24].

## 3. Results

### 3.1. Participants

A total of 201 healthy participants (132 females and 69 males) were included in the data analyses and were subdivided into three different age cohorts (67 participants per group). Their detailed characteristics, according to age and BMI, are presented in Table 1.

### 3.2. Data Analysis

**Figure 2:**
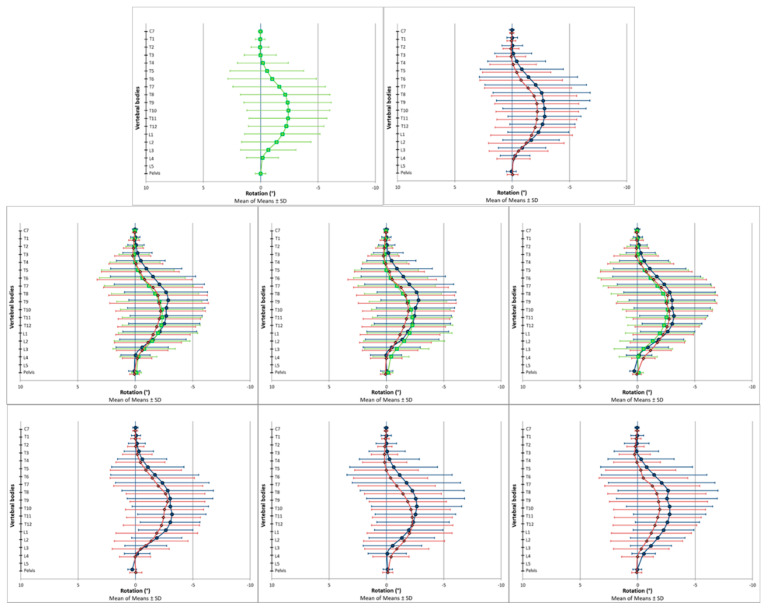
Vertebral body positions in the transversal plane. Positive values represent a rotation of the vertebral bodies to the left (counterclockwise), and negative values represent a rotation of the vertebral bodies to the right (clockwise). The scale of the *x*-axis is turned to enhance the intuitive visual interpretability of the results: (**Upper row**) (left picture: all participants (◼, green); right picture: all female (◆, red) and all male (●, blue) participants). (**Middle row**) (left picture: all participants of the respective age cohorts: young (●, blue), middle (◼, green) and old (◆, red); middle picture: all female participants of the respective age cohorts: young (●, blue), middle (◼, green) and old (◆, red); right picture: all male participants of the respective age cohorts: young (●, blue), middle (◼, green) and old (◆, red)). (**Lower row**) (left picture: all young female (◆, red) and all young male (●, blue) participants; middle picture: all middle-aged female (◆, red) and all middle-aged male (●, blue) participants; right picture: all old female (◆, red) and all old male (●, blue) participants).

**Figure 3:**
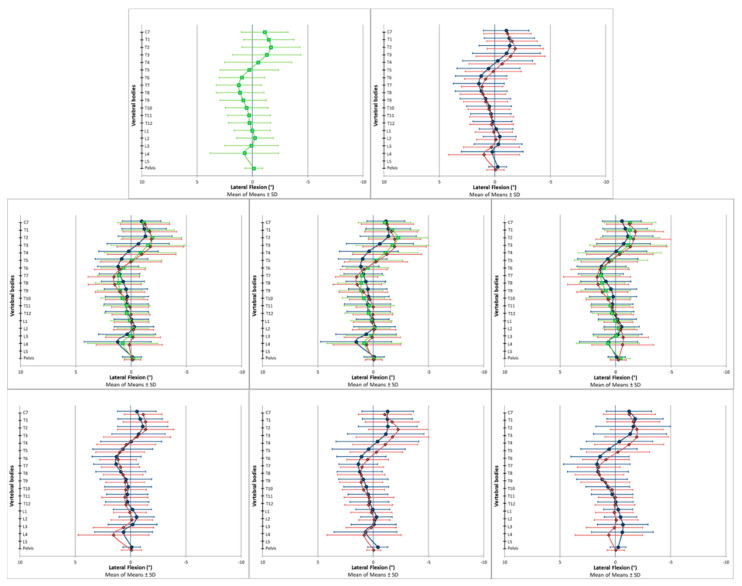
Vertebral body positions in the coronal plane. Positive values represent a tilt of the vertebral bodies to the left, and negative values represent a tilt of the vertebral bodies to the right. The scale of the *x*-axis is turned to enhance the intuitive visual interpretability of the results: (**Upper row**) (left picture: all participants (◼, green); right picture: all female (◆, red) and all male (●, blue) participants). (**Middle row**) (left picture: all participants of the respective age cohorts: young (●, blue), middle (◼, green) and old (◆, red); middle picture: all female participants of the respective age cohorts: young (●, blue), middle (◼, green) and old (◆, red); right picture: all male participants of the respective age cohorts: young (●, blue), middle (◼, green) and old (◆, red)). (**Lower row**) (left picture: all young female (◆, red) and all young male (●, blue) participants; middle picture: all middle-aged female (◆, red) and all middle-aged male (●, blue) participants; right picture: all old female (◆, red) and all old male (●, blue) participants).

**Figure 4:**
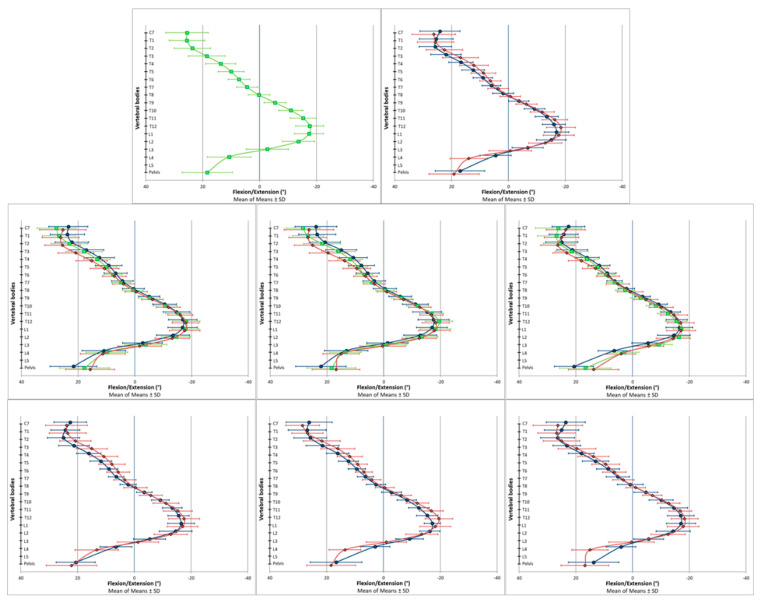
Vertebral body positions in the sagittal plane. Positive values represent a tilt of the vertebral bodies towards spinal flexion, and negative values represent a tilt of the vertebral bodies towards spinal extension. The scale of the *x*-axis is turned to enhance the intuitive visual interpretability of the results: (**Upper row**) (left picture: all participants (◼, green); right picture: all female (◆, red) and all male (●, blue) participants). (**Middle row**) (left picture: all participants of the respective age cohorts: young (●, blue), middle (◼, green) and old (◆, red); middle picture: all female participants of the respective age cohorts: young (●, blue), middle (◼, green) and old (◆, red); right picture: all male participants of the respective age cohorts: young (●, blue), middle (◼, green) and old (◆, red)). (**Lower row**) (left picture: all young female (◆, red) and all young male (●, blue) participants; middle picture: all middle-aged female (◆, red) and all middle-aged male (●, blue) participants; right picture: all old female (◆, red) and all old male (●, blue) participants.

The spinal posture data were analyzed using descriptive and explorative statistics. Reference values for the specific and global spine parameters are presented in Table 2 for the transversal plane, in Table 3 for the coronal plane and in Table 4 for the sagittal plane. Figure 2 (transversal), Figure 3 (coronal) and Figure 4 (sagittal) are the respective graphical representations of the specific spine parameters for those three investigated planes. The results of the explorative statistical analyses are presented in Table 5.

#### 3.2.1. Descriptive Data Analysis

In the transverse plane, the spine was not in a neutral rotary position. Instead, a systematic vertebral rotation to the right side was identified from T5 to L3 among all the investigated subgroups (Figure 2 and Table 2). In the coronal plane, a systematic deviation from the neutral centerline was also apparent. The vertebrae above T5 were laterally flexed to the right side, and around the fifth thoracic vertebrae, the side of lateral flexion changed in direction to the left (Figure 3 and Table 3). In the sagittal plane, T8 was found to be in an almost neutral position, indicating that it was the thoracic kyphosis apex (Figure 4 and Table 4). The vertebrae above (C7–T7) were tilted towards spinal flexion, while the vertebrae below were positioned in spinal extension (T9–~L3). The height of the lumbar lordosis apex, meaning the reverse change in direction from spinal extension to spinal flexion, differed between the analyzed subgroups but was systematically located between L2 and L4.

The graphical data output of the specific spine parameters indicated systematic differences between the female and male participants and between the participants in the different age cohorts. In the transverse plane, these subgroup-dependent visual differences were present among almost all the investigated vertebral bodies. In the coronal plane, the differences seemed to be locally limited to the upper thoracic spine. In the sagittal plane, the curves of the analyzed subgroups ran more in parallel compared to the other two planes. In this regard, the differently scaled x-axes have to be considered.

#### 3.2.2. Explorative Data Analysis

The graphically apparent deviations of the vertebral bodies from the symmetrical zero position in the transverse and the coronal planes could be confirmed by statistical data analyses. The deviations were significant from T5 to L3 in the transverse plane and from C7 to T4, from T6 to T12 and for the pelvis in the coronal plane when that data of the entire group were considered and tested versus a hypothetical median of zero. Likewise, all the global parameters in the two respective planes deviated significantly from the respective symmetrical spine position (Table 5). The visual differences between the analyzed subgroups, however, could not be statistically confirmed for the transverse and coronal plane data. Here, only the isolated parameters revealed statistical trends pointing towards a possible existing difference (for “Pelvis Rotation” between the young and middle participants (*p* = 0.05) and for “Right Side Apical Deviation VP-DM + max (mm)” (*p* = 0.04) between the female and male participants).

In the sagittal plane, systematic deviations from a straight upright spine position existed in all the vertebral bodies except for T8 (neutral vertebrae of the thoracic kyphosis) and all the global spine parameters. In contrast with the two other planes, the statistical analyses also revealed systematic trends pointing towards possible differences between the analyzed subgroups. The global parameter of “Lumbar Lordosis (ITL-ILS) (°)” differed between the female and male participants (*p* < 0.001), while the parameter of “Thoracic Kyphosis (ICT-ITL) (°)” indicated a trend towards a difference between the participants in the different age cohorts (*p* < 0.001). The systematic trend behind these findings becomes apparent when observing the specific spine parameters. Sex-specific differences could be found for all the specific parameters except for the two major turning points (meaning the most flexed (T1) and the most extended (L1) vertebrae). Differences between the age cohorts in the global parameter of “Thoracic Kyphosis (ICT-ITL) (°)” were also apparent at the level of the specific spine parameters. The systematic differences due to the participants’ belonging to different age cohorts can be seen here in the isolated upper thoracic vertebrae (C7–T4) and the pelvis (Table 5).

### 3.3. Literature Comparison

Table 6 compares the results for the global spine parameters of the current study with those derived from previous publications using the same VRS measurement device [18,19,20,21,22,23]. Most of the results were found to be almost comparable; however, there was a trend towards slightly lower values derived from the current study for the parameters of the transverse and the coronal plane when compared to those of previous research.

## 4. Discussion

Various studies have analyzed spinal posture and its possible adaptations to different spinal and other musculoskeletal pathologies using VRS [29,30,31]. However, reference values for the comparison of the possible pathological findings are only available for global spine parameters that mainly derived from children [18], younger adults [19,20,21], or young and middle-aged adults, but these are based on very small numbers of participants [22,23]. Systematically collected normative data, which differentiates between subgroups according to sex and age, which can be used to identify possible changes in spinal posture over the adult life span, were missing. One aim of this study was, therefore, to complement existing knowledge with a further reference dataset that meets those requirements. Spinal posture data were thus captured and analyzed based on 201 healthy participants according to sex and age over an adult life span of 18 to 70 years. A further aim was to expand the current knowledge by providing an additional reference dataset of specific spine parameters that contains three-dimensional posture data for every vertebral body (from C7 to L4 and the pelvis).

### 4.1. Global Spine Parameters

The results for the global spine parameters derived from the current study did not differ greatly from those of previous publications using the same VRS measurement device ([19,20,21,22,23]; Table 6). However, there seems to be a trend towards slightly lower measurement results for the parameters in the transverse and the coronal planes. Possible explanations for the deviation of the results could be, in addition to the different cohort compositions and cohort sizes, differences in the measurement protocol and data analysis. To obtain the most accurate data quality, we used a high standardization of the measurement protocol, the use of additional markers in the course of the vertebral column (~T3 and ~T12) and the systematic removal of extreme outliers from the raw dataset. Since the comparative studies do not provide corresponding information, the question regarding the reasons for the differences cannot be answered in a well-established manner.

In the current study, significant trends towards possible differences in several global spine parameters according to sex and age cohort were revealed through explorative data analyses. While these differences were not found to be systematic for the coronal plane parameter of the “(Right Side) Apical Deviation VP-DM + max (mm)”, the results for the respective sagittal plane parameters were considered highly important. The “Lumbar Lordosis (ITL-ILS) (°)” angle revealed a trend towards a significant difference between the female and male participants, with females showing greater lordosis angles than their male counterparts. This is in accordance with previous publications using the same VRS measuring device [19,20,21]. These findings also match the results of a recent systematic review and meta-analysis describing age- and sex-based effects on the lumbar lordosis angles and the range of motion based on different clinically established measurement approaches (radiological and non-radiological). The authors also found significant differences according to sex, with females having greater lumbar lordosis angles than men, but in contrast to the current findings, they also revealed indications that age possibly affected the respective spine parameters [32]. Similar correlations between VRS- and X-ray-measured results were found for the sagittal plane parameter of “Thoracic Kyphosis”. The current study found a trend towards significant differences between young and old (*p* < 0.001) and between middle and old (*p* < 0.02) participants, indicating an increase in the VRS-measured parameter with increasing age. Comparable results were published in a recent systematic review based on radiography-based Cobb angle calculations [33]. The authors described an increase in thoracic kyphosis with aging but did not find that sex affected the spine parameters.

These results confirm the importance of having VRS reference data that are not only distinguishable between subgroups according to sex, as has been the case thus far, but also according to different age cohorts. Without these data, changes in spinal posture that physiologically occur over a healthy life span could be falsely diagnosed as pathologic, resulting in troubling uncertainty for the affected patients in clinical practice.

### 4.2. Specific Spine Parameters

Apart from a previous sub-analysis of 100 female participants from the present study cohort, which was published by our own research group [24], this is the first paper to present systematic reference data for every vertebral body in all three dimensions for both sexes and for three different age cohorts, which were derived using the VRS “Formetric III 4D” measuring device. As already described, in contrast to the currently common clinical beliefs, the physiological spinal posture in the transverse and the coronal planes was not found to be straight and symmetric with regard to rotation and lateral flexion [24]. Instead, a systematic rotation of the mid- and lower thoracic and lumbar vertebrae towards the right side was observed and supported by explorative data analyses. This rotation was found to be more pronounced in males than females and in young compared to middle-aged and old participants. A pre-existing vertebral rotation in healthy participants was also previously described based on CT and MRI measurements [34]. In patients with a diagnosed situs inversus totalis, the side of the vertebral rotation changed, respectively [35]. These results suggest that the VRS findings of the current study are clinically comprehensible and that internal organ arrangement might be a possible physiological cause of the observed asymmetric spinal posture.

In the coronal plane, a lateral flexion to the right side was found in the case of the upper thoracic vertebrae, while the underlying vertebrae showed a systematic lateral flexion to the left. Contrary to the results for the spinal rotation, visually, the lateral flexion in the upper thoracic vertebrae seemed to be more pronounced in the female compared to the male participants, whereas no sex-specific differences were detectable in the mid- and lower thoracic or the lumbar vertebral body positions. According to age-related differences, the younger group visually seemed to demonstrate less lateral flexion than the middle-aged and old participants, specifically in the thoracic spinal region. Whether or not this might be caused by posture adaptations induced by normal degenerative changes in the spine remains unclear. However, Kilshaw et al. [36], who analyzed the lumbar spine retrospectively based on abdominal radiographs and found that deformities such as lumbar scoliosis, lateral listhesis and osteoarthritis in the coronal plane started to occur after the age of 50 and steadily increased with age, previously described such an effect, albeit in a different spinal section.

No unexpected outcomes for the vertebral positions were detected in the sagittal plane parameters. The apex of the VRS-measured thoracic kyphosis, meaning the least tilted vertebrae, appeared around T8. This is in accordance with recently published findings derived from radiographic data. Here, the thoracic apex was located between T7 and T9 [37,38]. In the current study, the lumbar lordosis apex appeared between the second and the fourth lumbar vertebrae, depending on the analyzed subgroup. As already described, a statistically significant trend towards a difference between the female and male participants was found for the global parameter of “Lumbar Lordosis (ITL-ILS) (°)”. This difference according to sex was also apparent among almost all the specific spine parameters, except for the two major curvature turning points (the most flexed (T1) and the most extended (L1) vertebral body). In the thoracic spine, males showed a greater curvature in the upper thoracic spine, and females showed greater curvature in the lower thoracic spine. The authors assume that this difference between females and males canceled each other out, which is why the sex difference did not manifest in the global variable of “Thoracic Kyphosis (ICT-ITL) (°)”. Nevertheless, the global parameter of “Thoracic Kyphosis (ICT-ITL) (°)” revealed significant differences between the analyzed age cohorts caused by the significant age differences in the respective specific parameters of the upper thoracic spine. Sex differences in spinopelvic alignment and in per-level vertebral inclination have also been reported in healthy participants based on upright low-dose digital biplanar X-ray analyses [39]. Similar to the current study, more dorsally inclined vertebrae were found in females than in males from T1 down to L2. In the current study, females showed more extended vertebral positions from T2 to L3 compared to their male counterparts. Even though the isolated raw values for vertebral inclination differed slightly between the two measurement approaches, the functional comparability of our results with those derived from X-ray analyses further supports the clinical importance of VRS as a non-invasive and simultaneously quick and easy assessment tool for spinal posture analysis in daily clinical routines.

However, despite the described functional agreement between the results based on the VRS and X-ray based measurements, the visual differences in the graphical data outputs between the analyzed subgroups could not be confirmed through statistical analyses for the specific spine parameters in the transverse or the coronal plane in the current study. One reason for this might be the large standard deviation identified in each of the analyzed variables. A normal spine anatomy also means that the results for the parameters in the transverse and the coronal planes are distribute naturally in a preferably narrow corridor reflecting an almost neutral spine position. No physiologically large differences are expected. Due to the high individuality displayed in the large standard deviation and those additional anatomical conditions, the analyzed sample of 201 healthy participants (and, partly, less than *n* = 67 in the respective subgroups) might have simply been too small to detect potential significant differences in the respective planes.

The fact that, in contrast, statistically significant trends in the possible differences between the analyzed subgroups were revealed for the parameters in the sagittal plane might be due to the presence of the two physiological major spinal curvatures, “thoracic kyphosis” and “lumbar lordosis”. These anatomical conditions mean that there is a higher natural deviation from the neutral position throughout the whole vertebral column, making it easier to detect statistically significant deviations between the analyzed subgroups even in this “small” sample of 201 healthy participants.

The possibility that sagittal plane parameters will be suitable for detecting differences between subgroups and between different pathologies is in accordance with previously published research [40,41]. Artificial intelligence (AI)-driven analyses of VRS measurement results also found sagittal plane parameters to be one of the most important features with which to distinguish pathology-independent spinal posture data from healthy comparative datasets [41]. Similar results were found when dynamic VRS gait data were analyzed by AI-driven methods. Here, the parameters of the coronal and the sagittal planes were most relevant for the classifications between the sexes [40]. Whether or not sagittal spine parameters have the potential to systematically distinguish between physiological and pathological spinal postures and which parameters are specifically involved must be investigated further in the future. Nevertheless, the first results point in this direction.

### 4.3. Limitations

The manufacturer of the “Formetric III 4D” system recommends the use of reflective markers for spinal posture analysis only when the software is not able to identify the required visual landmarks (vertebra prominens (VP) and the two lumbar dimples (DM)) on its own. However, the use of three reflective markers for the landmarks is necessary for dynamic gait analysis. As this study is part of an overarching research project aiming to collect reference data for spinal posture in the habitual stance and when walking at four different walking speeds among the same healthy study cohort, it was necessary to mark all the participants with the three markers in order to render the stance and gait results comparable with each other. Software misinterpretations that arose in advance during the test measurements at the fast walking speeds, caused by the soft tissue and scapular motions of the participants’ back surface, meant that the researchers decided to use two additional markers (~T3 and ~T12) to stabilize the systems’ dynamic data analysis procedures. The researchers also decided to mark C7 and the PSIS instead of the VP and DM. This approach was chosen because marking C7 and the PSIS is recommended in cases where the VP and DM are not clearly identifiable on the surface of the participant’s back. In order to standardize the measuring procedure and to render the results as comparable to each other as possible, the marking of C7 and the PSIS was determined a priori for all the participants, even though this technique was definitely more prone to palpation bias [42,43]. Furthermore, only participants with a BMI of ≤30.0 kg/m^2^ were included in the study due to data capture requirements. The procedures described enabled the collection of highly standardized data under controlled laboratory conditions. Nevertheless, the researchers are aware that this approach limits the external validity of the presented findings and, thus, their direct transferability to clinical practice.

The large number of parameters analyzed and the resulting high number of tested hypotheses also mean that the significant results have to be interpreted with caution. They are more suitable for showing trends in possible differences rather than real statistical significance. In this regard, it must also be mentioned that, retrospectively, the chosen sample size seemed to be too small to detect potential differences between the analyzed subgroups, especially for the specific spine parameters.

Finally, yet importantly, their radiation and contact-free nature mean that results derived from VRS measurements are calculated and based on mathematical algorithms. Even though their validity has been investigated in various publications, those studies mainly focused on comparisons between X-ray and VRS data captured from patients affected by different spinal pathologies (mainly scoliosis) [8,14,15,44,45]. For ethical reasons, no such comparative studies based on healthy participants are available. The results presented here, however, reveal a strong functional agreement with the results derived from clinically established measurement approaches, such as X-ray or MRI/CT scans, and VRS measurements [33,34,39]. This underlines the potential of VRS to serve as a non-invasive, quick and objective alternative for spinal posture analysis in clinical practice, especially when the therapeutic focus lies in function-orientated clinical outcomes and when pre-post measurements are required.

## 5. Conclusions

This study complements the existing VRS reference datasets for global spine parameters by adding normative values for different subgroups according to sex and age over an adult life span from 18 to 70 years. The closure of this gap, retrospectively, was found to be very important, because relevant changes over the life span in the isolated spine parameters became visible. Reference values for the specific spine parameters of every vertebral body from C7 to L4 in all three dimensions according to sex and age were presented and revealed visual but statistically non-significant differences between the analyzed subgroups. The sagittal plane parameters seem to have the greatest potential to detect differences between groups of participants. Whether or not those variations are possibly significant must be investigated in future studies by repeating the current project with an appropriate number of healthy participants.

The great variation in and individuality of the spinal posture displayed in the large standard deviation of the analyzed parameters, which was described previously by our research group using data derived from VRS measurements of asymptomatic female volunteers [24], were confirmed for the respective subgroups in the current study. Most importantly, and against widespread clinical expectations, the healthy human spine was found to be systematically asymmetric in the transverse and the coronal planes during upright habitual standing. There needs to be discussion in the therapeutic setting about whether approximations to an almost symmetrical spine in the respective planes are biomechanically desirable in any way [24].

## Figures and Tables

**Table 1:** Participant characteristics.

		All Participants	Sex		Age Cohort “Young”			Age Cohort “Middle”			Age Cohort “Old”		
			All Females	All Males	All Young Participants	Young Females	Young Males	All Middle Participants	Middle Females	Middle Males	All Old Participants	Old Females	Old Males
**N**		201	132	69	67	44	23	67	44	23	67	44	23
**Age (years)**	Mean	41.3	41.3	41.3	25.9	26.0	25.6	41.4	42.2	39.8	56.6	55.7	58.3
	SD	13.4	13.0	14.3	2.9	2.7	3.3	6.4	6.5	6.2	4.3	3.9	4.5
**BMI (kg/m^2^)**	Mean	23.5	22.9	24.6	22.7	22.0	23.9	23.7	23.0	25.0	24.1	23.6	25.0
	SD	2.8	2.8	2.4	2.9	2.9	2.6	2.8	2.8	2.3	2.5	2.5	2.2

**Table 2:** Results of the descriptive statistical analyses (MoM ± SD) of the spinal parameters in the transversal plane.

			Specific Parameters																			Global Parameters			
			Sh (°)	C7 (°)	T1 (°)	T2 (°)	T3 (°)	T4 (°)	T5 (°)	T6 (°)	T7 (°)	T8 (°)	T9 (°)	T10 (°)	T11 (°)	T12 (°)	L1 (°)	L2 (°)	L3 (°)	L4 (°)	Pel (°)	Surface Rotation RMS (°)	Surface Rotation MAX (°)	(Right Side) Surface Rotation +Max (°)	(Left Side) Surface Rotation −Max (°)
**All Participants**		N	198	199	201	200	200	197	197	198	198	198	200	201	201	201	201	200	200	200	200	200	201	198	201
		MoM	−0.3	0.0	0.0	0.1	0.0	−0.2	−0.5	−1.0	−1.6	−2.1	−2.3	−2.4	−2.4	−2.2	−1.9	−1.4	−0.7	−0.2	0.0	2.3	−1.9	1.5	−3.3
		SD	2.0	0.2	0.4	0.8	1.4	2.2	3.2	3.9	4.0	3.9	3.8	3.6	3.4	3.3	3.3	3.0	2.4	1.4	0.5	0.9	4..0	1.4	2.3
**Sex**	**All Females**	N	130	131	132	131	131	129	129	130	130	130	131	132	132	132	132	131	131	131	132	131	132	130	132
		MoM	−0.3	0.0	0.1	0.1	0.1	−0.1	−0.4	−0.8	−1.4	−1.9	−2.2	−2.2	−2.1	−2.0	−1.7	−1.2	−0.6	−0.1	0.0	2.2	−1.8	1.5	−3.2
		SD	2.0	0.2	0.4	0.7	1.3	2.0	3.0	3.6	3.8	3.7	3.7	3.6	3.5	3.5	3.6	3.3	2.6	1.4	0.5	0.9	4.0	1.4	2.3
	**All Males**	N	68	68	69	69	69	68	68	68	68	68	69	69	69	69	69	69	69	69	68	69	69	68	69
		MoM	−0.3	0.0	0.0	0.0	−0.1	−0.4	−0.8	−1.4	−2.1	−2.6	−2.7	−2.8	−2.8	−2.6	−2.3	−1.6	−0.9	−0.2	0.1	2.3	−2.1	1.7	−3.6
		SD	2.0	0.2	0.5	0.9	1.6	2.5	3.6	4.3	4.4	4.2	4.1	3.6	3.2	2.8	2.7	2.5	2.1	1.3	0.4	0.9	4.0	1.4	2.3
**Age Cohort** **“Young”**	**All Young** **Participants**	N	67	67	67	67	67	67	67	67	67	66	66	67	67	67	67	66	66	66	67	67	67	66	67
		MoM	−0.1	0.0	0.0	−0.1	−0.2	−0.5	−1.0	−1.5	−2.1	−2.7	−2.9	−2.7	−2.7	−2.5	−2.1	−1.5	−0.6	0.0	0.1	2.3	−2.3	1.4	−3.5
		SD	2.1	0.2	0.4	0.7	1.3	2.1	3.1	3.7	3.9	3.6	3.4	3.4	3.1	3.1	3.2	2.9	2.3	1.3	0.5	0.9	3.6	1.2	2.2
	**Young** **Females**	N	44	44	44	44	44	44	44	44	44	43	43	44	44	44	44	43	43	43	44	44	44	43	44
		MoM	0.1	0.0	0.0	0.0	−0.2	−0.4	−0.9	−1.5	−2.0	−2.6	−2.8	−2.5	−2.4	−2.3	−1.9	−1.4	−0.5	0.0	0.0	2.4	−2.4	1.3	−3.5
		SD	2.0	0.2	0.4	0.7	1.3	2.1	3.1	3.7	3.9	3.4	3.3	3.4	3.2	3.3	3.6	3.2	2.5	1.4	0.5	0.9	3.8	1.3	2.3
	**Young** **Males**	N	23	23	23	23	23	23	23	23	23	23	23	23	23	23	23	23	23	23	23	23	23	23	23
		MoM	−0.4	0.0	0.0	−0.1	−0.3	−0.6	−1.1	−1.7	−2.3	−2.8	−3.0	−3.0	−3.2	−3.0	−2.6	−1.9	−0.9	−0.2	0.3	2.2	−2.3	1.7	−3.6
		SD	2.2	0.2	0.4	0.7	1.3	2.1	3.2	3.8	4.1	4.0	3.7	3.3	3.0	2.6	2.4	2.2	1.8	1.1	0.4	0.8	3.4	1.2	2.2
**Age Cohort** **“Middle”**	**All Middle** **Participants**	N	66	67	67	67	67	67	67	67	67	67	67	67	67	67	67	67	67	67	67	67	67	67	67
		MoM	−0.5	0.0	0.1	0.1	0.1	0.0	−0.2	−0.6	−1.2	−1.7	−2.1	−2.3	−2.3	−2.3	−2.0	−1.5	−0.8	−0.3	−0.1	2.1	−2.3	1.4	−3.4
		SD	2.1	0.2	0.4	0.7	1.3	2.2	3.2	3.7	3.9	3.8	3.7	3.6	3.5	3.4	3.5	3.3	2.7	1.6	0.4	1.0	3.6	1.4	2.2
	**Middle** **Females**	N	43	44	44	44	44	44	44	44	44	44	44	44	44	44	44	44	44	44	44	44	44	44	44
		MoM	−0.4	0.0	0.1	0.1	0.2	0.2	0.0	−0.4	−0.9	−1.4	−1.9	−2.1	−2.3	−2.2	−2.0	−1.5	−0.9	−0.4	−0.1	2.0	−2.2	1.3	−3.3
		SD	2.1	0.2	0.3	0.6	1.2	1.9	2.8	3.2	3.4	3.4	3.4	3.4	3.5	3.5	3.7	3.5	2.8	1.6	0.4	0.8	3.4	1.4	2.0
	**Middle** **Males**	N	23	23	23	23	23	23	23	23	23	23	23	23	23	23	23	23	23	23	23	23	23	23	23
		MoM	−0.6	0.0	0.0	0.0	−0.1	−0.3	−0.6	−1.1	−1.7	−2.3	−2.6	−2.6	−2.5	−2.3	−1.9	−1.3	−0.5	−0.1	−0.1	2.3	−2.4	1.5	−3.6
		SD	2.1	0.2	0.4	0.9	1.6	2.6	3.8	4.6	4.7	4.5	4.3	3.9	3.5	3.2	3.0	2.9	2.6	1.7	0.4	1.1	3.8	1.4	2.6
**Age Cohort** **“Old”**	**All Old** **Participants**	N	65	65	67	66	66	63	63	64	64	65	67	67	67	67	67	67	67	67	66	66	67	65	67
		MoM	−0.3	0.0	0.1	0.1	0.2	−0.1	−0.4	−0.8	−1.5	−2.0	−2.1	−2.2	−2.1	−1.9	−1.6	−1.1	−0.6	−0.2	0.1	2.3	−1.1	1.8	−3.1
		SD	2.0	0.2	0.5	0.9	1.6	2.3	3.4	4.1	4.3	4.3	4.3	3.9	3.6	3.4	3.2	2.9	2.2	1.3	0.4	0.9	4.6	1.7	2.4
	**Old** **Females**	N	43	43	44	43	43	41	41	42	42	43	44	44	44	44	44	44	44	44	44	43	44	43	44
		MoM	−0.6	0.0	0.1	0.2	0.3	0.1	−0.3	−0.5	−1.3	−1.7	−1.8	−1.9	−1.7	−1.5	−1.2	−0.8	−0.3	0.0	0.1	2.3	−0.9	1.8	−2.9
		SD	2.0	0.2	0.4	0.8	1.4	2.0	3.1	3.9	4.1	4.3	4.3	4.0	3.8	3.6	3.5	3.1	2.4	1.4	0.4	0.9	4.6	1.7	2.4
	**Old** **Males**	N	22	22	23	23	23	22	22	22	22	22	23	23	23	23	23	23	23	23	22	23	23	22	23
		MoM	0.3	0.1	0.0	0.1	0.1	−0.3	−0.8	−1.4	−2.1	−2.6	−2.5	−2.8	−2.8	−2.6	−2.2	−1.7	−1.2	−0.5	0.0	2.5	−1.4	1.8	−3.5
		SD	1.8	0.2	0.6	1.1	1.9	2.9	4.0	4.6	4.6	4.3	4.4	3.8	3.1	2.8	2.6	2.4	1.8	1.0	0.4	0.9	4.8	1.7	2.3

Abbreviations: MoM = mean of means; SD = standard deviation; Sh = shoulder; Pel = pelvis; N = number.

**Table 3:** Results of the descriptive statistical analyses (MoM ± SD) of the spinal parameters in the coronal plane.

			Specific Parameters																			Global Parameters					
			Sh (°)	C7 (°)	T1 (°)	T2 (°)	T3 (°)	T4 (°)	T5 (°)	T6 (°)	T7 (°)	T8 (°)	T9 (°)	T10 (°)	T11 (°)	T12 (°)	L1 (°)	L2 (°)	L3 (°)	L4 (°)	Pel (°)	Trunk Imbalance VP-DM (°)	Trunk Imbalance VP-DM (mm)	Apical Deviation RMS (mm)	Apical Deviation MAX (mm)	(Right Side) Apical Deviation VP-DM +Max (mm)	(Left Side) Apical Deviation VP-DM −Max (mm)
**All Participants**		N	200	197	195	194	199	198	198	198	201	201	200	200	200	199	198	198	198	200	194	194	195	196	201	198	198
		MoM	−1.1	−1.1	−1.5	−1.7	−1.3	−0.5	0.3	1.0	1.2	1.1	0.8	0.5	0.3	0.3	0.0	−0.2	0.1	0.7	−0.1	−0.2	−1.7	3.6	−1.8	3.2	−4.9
		SD	1.3	2.1	2.3	2.6	3.1	3.0	2.6	2.1	2.0	2.2	2.1	1.9	1.9	1.9	1.6	1.7	2.4	3.1	0.8	0.8	7.3	1.7	7.1	3.0	3.3
**Sex**	**All Females**	N	131	129	128	127	132	131	131	131	132	132	131	131	131	130	129	129	129	131	127	126	128	130	132	131	130
		MoM	−1.3	−1.2	−1.6	−1.8	−1.4	−0.7	0.1	0.8	1.1	1.1	0.8	0.5	0.3	0.3	0.1	−0.1	0.3	1.0	−0.1	−0.2	−1.6	3.5	−2.3	2.9	−4.9
		SD	1.4	2.2	2.3	2.5	3.1	3.0	2.5	1.9	1.9	2.1	2.0	1.9	2.0	2.0	1.7	1.8	2.5	3.2	0.8	0.9	7.7	1.7	6.7	2.8	3.3
	**All Males**	N	69	68	67	67	67	67	67	67	69	69	69	69	69	69	69	69	69	69	67	68	67	66	69	67	68
		MoM	−0.9	−1.0	−1.3	−1.3	−1.1	−0.3	0.6	1.2	1.4	1.2	0.8	0.5	0.4	0.2	−0.1	−0.4	−0.3	0.2	−0.3	−0.2	−2.0	3.8	−0.8	3.8	−4.7
		SD	1.3	2.1	2.3	2.8	3.1	3.2	2.8	2.3	2.3	2.4	2.3	2.0	1.8	1.8	1.5	1.5	2.2	2.8	0.8	0.8	6.6	1.6	7.7	3.4	3.2
**Age Cohort** **“Young”**	**All Young** **Participants**	N	67	66	66	66	67	66	66	66	67	67	67	67	67	67	67	67	66	66	64	66	66	65	67	66	66
		MoM	−1.0	−0.9	−1.2	−1.3	−0.6	0.2	0.9	1.2	1.0	0.7	0.5	0.4	0.4	0.4	0.0	−0.3	0.4	1.2	−0.1	−0.3	−2.5	3.5	−0.9	3.3	−4.4
		SD	1.2	1.7	2.0	2.4	2.8	2.7	2.4	1.9	1.8	1.9	1.9	2.0	2.0	1.9	1.6	1.8	2.6	3.0	0.9	0.8	6.5	1.6	7.0	2.9	3.1
	**Young** **Females**	N	44	43	43	43	44	43	43	43	44	44	44	44	44	44	44	44	43	43	42	43	43	42	44	43	43
		MoM	−1.1	−1.1	−1.4	−1.4	−0.6	0.4	0.9	1.1	0.9	0.7	0.5	0.4	0.5	0.4	0.1	−0.1	0.6	1.5	−0.1	−0.3	−2.6	3.4	−1.1	3.1	−4.4
		SD	1.3	1.7	2.0	2.5	3.0	2.7	2.2	1.7	1.7	1.8	1.7	1.9	2.1	2.0	1.5	1.9	2.7	3.2	0.9	0.8	6.7	1.6	7.2	2.7	3.1
	**Young** **Males**	N	23	23	23	23	23	23	23	23	23	23	23	23	23	23	23	23	23	23	22	23	23	23	23	23	23
		MoM	−0.7	−0.6	−0.9	−1.1	−0.7	0.0	0.7	1.3	1.3	0.9	0.4	0.2	0.3	0.3	−0.2	−0.5	−0.2	0.6	−0.1	−0.2	−2.3	3.6	−0.4	3.8	−4.4
		SD	1.1	1.8	2.0	2.3	2.4	2.8	2.7	2.2	2.0	2.3	2.3	2.1	1.9	2.0	1.7	1.6	2.2	2.6	0.8	0.7	6.3	1.6	6.7	3.1	3.1
**Age Cohort** **“Middle”**	**All Middle** **Participants**	N	67	65	63	63	67	67	67	67	67	67	66	66	66	65	66	65	66	67	64	66	66	66	67	66	66
		MoM	−1.2	−1.1	−1.6	−1.9	−1.5	−0.8	0.0	0.7	1.1	1.1	1.0	0.8	0.4	0.4	0.1	−0.2	0.1	0.8	−0.2	−0.2	−1.9	3.8	−2.9	2.9	−5.3
		SD	1.3	2.3	2.4	2.7	3.3	3.2	2.7	2.0	1.9	2.1	2.0	1.9	2.1	2.0	1.7	1.4	2.2	3.2	0.8	0.8	7.0	1.8	7.0	3.2	3.3
	**Middle** **Females**	N	44	42	41	41	44	44	44	44	44	44	43	43	43	42	43	42	43	44	41	43	43	44	44	44	43
		MoM	−1.3	−1.0	−1.7	−2.2	−1.7	−1.1	−0.3	0.5	1.0	1.1	1.1	0.9	0.4	0.4	0.2	−0.1	0.2	0.8	−0.1	−0.2	−1.8	3.6	−3.3	2.8	−5.3
		SD	1.4	2.4	2.4	2.7	3.3	2.9	2.4	1.9	2.0	2.1	1.9	1.9	2.3	2.2	1.9	1.4	2.3	3.3	0.7	0.9	7.4	1.9	6.4	2.9	3.3
	**Middle** **Males**	N	23	23	22	22	23	23	23	23	23	23	23	23	23	23	23	23	23	23	23	23	23	22	23	22	23
		MoM	−1.0	−1.3	−1.3	−1.3	−1.1	−0.4	0.4	1.1	1.3	1.2	0.9	0.6	0.5	0.3	0.0	−0.3	0.0	0.7	−0.4	−0.2	−2.2	4.1	−2.2	3.2	−5.4
		SD	1.1	2.3	2.3	2.7	3.5	3.7	3.3	2.2	1.8	2.0	2.2	2.0	1.8	1.8	1.5	1.4	2.0	2.8	0.9	0.7	6.2	1.7	8.0	3.7	3.4
**Age Cohort** **“Old”**	**All Old** **Participants**	N	66	66	66	65	65	65	65	65	67	67	67	67	67	67	65	66	66	67	66	62	63	65	67	66	66
		MoM	−1.2	−1.3	−1.7	−1.8	−1.7	−1.0	0.0	1.0	1.6	1.5	1.0	0.4	0.1	0.0	0.0	−0.2	−0.2	0.2	−0.1	−0.1	−0.8	3.6	−1.5	3.3	−4.8
		SD	1.4	2.2	2.4	2.7	3.0	3.1	2.8	2.3	2.3	2.4	2.2	1.9	1.7	1.7	1.6	1.8	2.5	3.0	0.8	0.9	8.3	1.6	7.3	3.1	3.3
	**Old** **Females**	N	43	44	44	43	44	44	44	44	44	44	44	44	44	44	42	43	43	44	44	40	42	44	44	44	44
		MoM	−1.3	−1.3	−1.6	−1.9	−1.9	−1.2	−0.2	0.8	1.5	1.4	0.9	0.3	0.0	0.0	0.1	−0.1	0.1	0.6	0.0	−0.2	−0.4	3.5	−2.4	2.8	−5.1
		SD	1.3	2.3	2.4	2.4	2.9	3.1	2.9	2.1	1.9	2.2	2.2	1.9	1.6	1.8	1.7	2.0	2.5	3.0	0.8	1.0	8.8	1.7	6.5	2.7	3.5
	**Old** **Males**	N	23	22	22	22	21	21	21	21	23	23	23	23	23	23	23	23	23	23	22	22	21	21	23	22	22
		MoM	−1.0	−1.2	−1.8	−1.6	−1.3	−0.4	0.6	1.4	1.7	1.6	1.2	0.7	0.3	0.0	−0.3	−0.5	−0.7	−0.6	−0.2	−0.1	−1.4	3.7	0.3	4.4	−4.3
		SD	1.6	2.0	2.5	3.4	3.3	3.0	2.5	2.6	3.0	2.8	2.3	2.0	1.9	1.6	1.3	1.5	2.3	2.8	0.7	0.9	7.4	1.6	8.5	3.6	3.0

Abbreviations: MoM = mean of means; SD = standard deviation; Sh = shoulder; Pel = pelvis; N = number.

**Table 4:** Results of the descriptive statistical analyses (MoM ± SD) of the spinal parameters in the sagittal plane.

			Specific Parameters																			Global Parameters			
			Sh (°)	C7 (°)	T1 (°)	T2 (°)	T3 (°)	T4 (°)	T5 (°)	T6 (°)	T7 (°)	T8 (°)	T9 (°)	T10 (°)	T11 (°)	T12 (°)	L1 (°)	L2 (°)	L3 (°)	L4 (°)	Pel (°)	Trunk Inclination (VP-DM) (°)	Trunk Inclination (VP-DM) (mm)	Thoracic Kyphosis (ICT-ITL) (°)	Lumbar Lordosis (ITL-ILS) (°)
**All Participants**		N	-	201	201	200	200	199	200	199	200	201	201	201	200	200	200	199	201	201	201	198	198	200	201
		MoM	-	25.5	25.5	23.6	18.6	13.7	10.0	7.2	4.4	0.2	−5.4	−11.0	−15.3	−17.6	−17.4	−13.7	−2.7	10.7	18.4	3.1	26.0	49.9	40.9
		SD	-	7.5	6.3	6.4	6.5	5.3	4.6	3.9	3.7	3.8	3.9	4.2	4.5	4.9	5.1	5.7	7.4	7.7	8.9	2.1	17.5	8.3	9.2
**Sex**	**All Females**	N	-	132	132	131	132	132	132	131	132	132	132	132	131	131	131	130	132	132	132	130	130	131	132
		MoM	-	26.2	25.6	22.5	16.9	12.1	8.8	6.3	3.6	−0.6	−6.3	−11.9	−16.3	−18.5	−17.7	−13.0	−0.6	13.9	19.1	3.1	25.6	50.1	44.0
		SD	-	7.6	6.4	6.4	6.3	5.1	4.4	3.8	3.7	3.6	3.7	4.2	4.5	5.1	5.5	5.8	7.5	6.6	8.9	2.1	17.6	8.2	8.5
	**All Males**	N	-	69	69	69	68	67	68	68	68	69	69	69	69	69	69	69	69	69	69	68	68	69	69
		MoM	-	24.1	25.4	25.7	21.9	16.7	12.4	9.0	6.0	1.9	−3.7	−9.1	−13.5	−16.0	−16.9	−15.1	−6.7	4.5	17.0	3.0	26.7	49.6	34.9
		SD	-	7.1	6.0	5.7	5.4	4.3	4.0	3.4	3.3	3.7	3.6	3.7	4.0	4.1	4.3	5.2	5.5	5.5	8.7	2.0	17.4	8.6	7.3
**Age Cohort** **“Young”**	**All Young** **Participants**	N	-	67	67	66	67	67	67	67	67	67	67	67	67	67	67	67	67	67	67	66	66	66	67
		MoM	-	23.4	23.7	22.2	17.1	12.5	9.2	6.8	4.4	0.5	−5.0	−10.5	−14.7	−16.9	−16.8	−13.4	−2.7	10.9	21.6	2.9	24.8	46.5	40.1
		SD	-	6.9	6.0	5.9	6.2	5.3	4.7	4.0	3.8	3.9	3.8	4.2	4.7	5.0	5.2	5.8	7.0	7.7	8.3	2.2	18.4	7.1	8.9
	**Young** **Females**	N	-	44	44	43	44	44	44	44	44	44	44	44	44	44	44	44	44	44	44	43	43	43	44
		MoM	-	23.9	23.4	20.7	15.0	10.7	7.9	5.7	3.3	−0.4	−5.8	−11.1	−15.3	−17.6	−17.0	−12.9	−1.3	13.2	22.1	2.8	22.6	46.9	43.0
		SD	-	7.4	6.5	5.5	5.5	4.9	4.6	4.0	3.9	4.1	4.0	4.6	5.1	5.5	5.4	5.9	7.3	7.7	8.9	2.1	17.5	6.9	8.7
	**Young** **Males**	N	-	23	23	23	23	23	23	23	23	23	23	23	23	23	23	23	23	23	23	23	23	23	23
		MoM	-	22.6	24.3	25.0	21.2	16.1	11.8	9.0	6.4	2.2	−3.5	−9.1	−13.4	−15.6	−16.5	−14.6	−5.4	6.5	20.7	3.3	28.9	45.8	34.5
		SD	-	5.8	5.1	5.7	5.4	4.3	3.8	3.2	2.7	2.9	2.8	3.1	3.5	3.7	4.7	5.8	5.6	5.6	6.9	2.2	19.7	7.7	6.5
**Age Cohort** **“Middle”**	**All Middle** **Participants**	N	-	67	67	67	67	66	66	66	66	67	67	67	67	67	67	66	67	67	67	67	67	67	67
		MoM	-	27.7	26.7	23.1	17.9	13.3	10.1	7.7	4.9	0.6	−5.1	−10.6	−15.3	−18.1	−17.9	−14.5	−3.8	9.9	17.8	3.2	27.0	49.7	41.0
		SD	-	6.8	5.9	6.5	6.4	4.7	3.7	3.2	3.3	3.6	3.9	4.2	4.5	4.9	4.7	5.2	7.5	7.4	8.8	2.0	17.0	7.7	9.0
	**Middle** **Females**	N	-	44	44	44	44	44	44	44	44	44	44	44	44	44	44	43	44	44	44	44	44	44	44
		MoM	-	28.5	26.7	21.7	16.0	11.9	9.0	6.9	4.1	−0.4	−6.3	−11.9	−16.8	−19.5	−18.2	−13.5	−1.0	13.5	18.4	3.2	26.3	49.3	43.7
		SD	-	6.0	5.5	6.5	6.0	4.4	3.4	3.0	3.3	3.3	3.6	4.0	4.2	4.9	5.4	5.6	7.1	5.6	8.6	2.1	17.3	7.6	9.0
	**Middle** **Males**	N	-	23	23	23	23	22	22	22	22	23	23	23	23	23	23	23	23	23	23	23	23	23	23
		MoM	-	26.1	26.8	25.7	21.4	16.1	12.3	9.4	6.3	2.6	−2.8	−8.2	−12.4	−15.4	−17.2	−16.3	−9.2	3.0	16.7	3.2	28.3	50.5	35.7
		SD	-	8.1	6.6	5.7	5.8	3.9	3.5	3.0	2.9	3.4	3.5	3.4	3.7	3.8	2.9	3.6	4.9	5.2	9.1	1.9	16.7	7.9	6.2
**Age Cohort** **“Old”**	**All Old** **Participants**	N	-	67	67	67	66	66	67	66	67	67	67	67	66	66	66	66	67	67	67	65	65	67	67
		MoM	-	25.4	26.1	25.5	20.8	15.2	10.7	7.2	3.9	−0.4	−6.2	−11.8	−16.0	−17.9	−17.6	−13.2	−1.7	11.3	15.7	3.1	26.1	53.4	41.6
		SD	-	8.2	6.5	6.3	6.3	5.5	5.1	4.2	4.0	3.9	3.8	4.2	4.4	4.8	5.4	6.1	7.7	7.9	8.6	2.1	17.4	8.7	9.7
	**Old** **Females**	N	-	44	44	44	44	44	44	43	44	44	44	44	43	43	43	43	44	44	44	43	43	44	44
		MoM	-	26.3	26.7	25.0	19.6	13.8	9.5	6.5	3.3	−1.1	−6.9	−12.7	−16.8	−18.3	−17.8	−12.6	0.4	15.0	16.8	3.4	27.8	54.0	45.3
		SD	-	8.7	6.7	6.5	6.7	5.5	4.9	4.1	3.8	3.4	3.4	4.0	4.1	4.8	5.6	6.0	8.0	6.4	8.4	2.2	18.2	8.7	7.7
	**Old** **Males**	N	-	23	23	23	22	22	23	23	23	23	23	23	23	23	23	23	23	23	23	22	22	23	23
		MoM	-	23.5	25.0	26.4	23.2	18.1	13.0	8.7	5.2	0.8	−4.7	−10.1	−14.6	−17.0	−17.1	−14.3	−5.7	4.1	13.7	2.6	22.8	52.4	34.4
		SD	-	6.9	6.1	6.0	4.9	4.5	4.7	4.1	4.1	4.5	4.2	4.2	4.8	4.7	5.1	6.0	5.3	5.2	8.9	1.8	15.5	9.0	9.1

Abbreviations: MoM = mean of means; SD = standard deviation; Sh = shoulder; Pel = pelvis; N = number.

**Table 5:** Results of the explorative statistical analyses (one-sample Wilcoxon signed rank test and two-way analyses of variance) of the spinal parameters in all three planes of movement.

		Specific Parameters																			Global Parameters					
Transversal Plane																										
		Sh (°)	C7 (°)	T1 (°)	T2 (°)	T3 (°)	T4 (°)	T5 (°)	T6 (°)	T7 (°)	T8 (°)	T9 (°)	T10 (°)	T11 (°)	T12 (°)	L1 (°)	L2 (°)	L3 (°)	L4 (°)	Pel (°)	Surface Rotation RMS (°)		Surface Rotation MAX (°)	(Right Side) Surface Rotation +Max (°)		(Left Side) Surface Rotation −Max (°)
**AP vs. HM = 0** (**One-Sample** **Wilcoxon** **Signed** **Rank Test**)	**Observed** **Median**	−0.12	0.06	0.15	0.06	−0.04	−0.27	−0.73	−1.03	−1.60	−2.21	−2.56	−2.60	−2.74	−2.35	−1.98	−1.23	−0.69	−0.18	0.03	2.11		−2.73	1.17		−3.22
	**Standardized Test Statistic**	−1.71	2.35	1.43	0.87	0.06	−1.22	−2.33	−3.52	−5.22	−6.80	−7.48	−7.93	−8.16	−7.96	−7.10	−5.73	−3.86	−1.95	0.81	12.26		−6.28	11.44		−12.14
	***p*-Value**	0.09	0.02 *	0.15	0.39	0.96	0.22	0.02 *	0.00 *	0.00 *	0.00 *	0.00 *	0.00 *	0.00 *	0.00 *	0.00 *	0.00 *	0.00 *	0.05	0.42	0.00 *		0.00 *	0.00 *		0.00 *
**Between-Subject Effects** (**Two-Way ANOVA**)	**Sex** (**Sig**)	0.84	0.82	0.29	0.38	0.41	0.37	0.37	0.27	0.27	0.27	0.34	0.26	0.19	0.19	0.23	0.36	0.40	0.53	0.17	0.45		0.71	0.35		0.37
	**Age Cohort** (**Sig**)	0.50	0.63	0.40	0.30	0.27	0.48	0.50	0.48	0.52	0.47	0.52	0.76	0.66	0.60	0.67	0.82	0.99	0.71	0.01 *	0.45		0.17	0.25		0.69
	Sex *Age **Cohort** (**Sig**)	0.15	0.92	0.97	0.99	0.98	0.94	0.92	0.87	0.93	0.85	0.92	0.95	0.83	0.69	0.62	0.57	0.37	0.23	0.06	0.49		0.94	0.71		0.84
	**Young vs.** **Old** (**Sig**)	0.87	0.77	0.43	0.34	0.26	0.63	0.74	0.63	0.82	0.68	0.56	0.71	0.57	0.49	0.57	0.81	1.00	0.92	1.00	1.00		0.16	0.33		0.56
	**Young vs. Middle** (**Sig**)	0.52	0.94	0.92	0.72	0.54	0.48	0.44	0.44	0.47	0.41	0.58	0.80	0.83	0.89	0.97	1.00	0.97	0.67	0.05 *	0.55		1.00	0.99		0.97
	**Middle vs. Old** (**Sig**)	0.93	0.97	1.00	1.00	0.95	1.00	0.97	0.99	0.94	0.97	1.00	0.99	0.91	0.77	0.73	0.88	0.97	0.96	0.07	0.47		0.18	0.22		0.71
**Coronal Plane**																										
		**Sh** (**°**)	**C7** (**°**)	**T1** (**°**)	**T2** (**°**)	**T3** (**°**)	**T4** (**°**)	**T5** (**°**)	**T6** (**°**)	**T7** (**°**)	**T8** (**°**)	**T9** (**°**)	**T10** (**°**)	**T11** (**°**)	**T12** (**°**)	**L1** (**°**)	**L2** (**°**)	**L3** (**°**)	**L4** (**°**)	**Pel** (**°**)	**Trunk** **Imbalance** (**VP-DM**) (**°**)	**Trunk** **Imbalance** (**VP-DM**) (**mm**)	**Apical** **Deviation** **RMS** (**mm**)	**Apical** **Deviation MAX** (**mm**)	(**Right Side**) **Apical** **Deviation** (**VP-DM**) **+max** (**mm**)	(**Left Side**) **Apical** **Deviation** (**VP-DM**) **−max** (**mm**)
**AP vs. HM = 0** (**One-Sample** **Wilcoxon** **Signed** **Rank Test**)	**Observed** **Median**	−1.08	−1.11	−1.55	−1.68	−1.38	−0.73	0.11	0.93	1.23	1.30	1.02	0.68	0.43	0.21	0.05	−0.29	0.04	0.51	−0.07	−0.24	−2.35	3.19	−3.30	2.70	−4.52
	**Standardized Test Statistic**	−9.52	−6.77	−7.89	−7.80	−5.52	−2.30	1.22	5.92	7.62	6.61	5.15	3.74	2.41	1.97	0.22	−1.82	0.38	2.91	−1.70	−3.54	−3.39	12.14	−3.54	11.34	−12.11
	***p*-Value**	0.00 *	0.00 *	0.00 *	0.00 *	0.00 *	0.02 *	0.22	0.00 *	0.00 *	0.00 *	0.00 *	0.00 *	0.02 *	0.05 *	0.83	0.07	0.70	0.00 *	0.09	0.00 *	0.00 *	0.00 *	0.00 *	0.00 *	0.00 *
**Between-Subject Effects** (**two-way ANOVA**)	**Sex** (**Sig**)	0.08	0.72	0.47	0.20	0.43	0.40	0.29	0.18	0.32	0.61	0.95	0.94	0.81	0.78	0.35	0.19	0.09	0.10	0.11	0.75	0.76	0.23	0.16	0.04 *	0.61
	**Age Cohort** (**Sig**)	0.47	0.56	0.35	0.42	0.19	0.15	0.23	0.51	0.38	0.18	0.24	0.49	0.67	0.49	0.81	0.91	0.47	0.13	0.62	0.58	0.53	0.48	0.26	0.58	0.25
	Sex *Age **Cohort** (**Sig**)	1.00	0.55	0.70	0.76	0.75	0.44	0.49	0.81	0.95	0.99	0.80	0.60	0.77	0.99	0.92	0.92	0.76	0.60	0.49	0.93	0.88	0.88	0.72	0.55	0.79
	**Young vs.** **Old** (**Sig**)	0.63	0.78	0.50	0.53	0.12	0.07	0.19	0.96	0.44	0.12	0.38	1.00	0.67	0.62	1.00	1.00	0.40	0.14	1.00	0.86	0.54	0.95	0.88	1.00	0.70
	**Young vs. Middle** (**Sig**)	0.57	0.94	0.73	0.41	0.26	0.12	0.14	0.45	1.00	0.56	0.34	0.52	1.00	1.00	0.95	0.98	0.82	0.79	0.88	1.00	1.00	0.60	0.23	0.74	0.20
	**Middle vs. Old** (**Sig**)	1.00	0.98	0.98	1.00	0.97	0.99	1.00	0.75	0.66	0.61	1.00	0.63	0.73	0.59	0.94	1.00	0.78	0.57	0.95	1.00	1.00	0.90	0.48	0.77	0.64
**Sagittal Plane**																										
		**Sh** (**°**)	**C7** (**°**)	**T1** (**°**)	**T2** (**°**)	**T3** (**°**)	**T4** (**°**)	**T5** (**°**)	**T6** (**°**)	**T7** (**°**)	**T8** (**°**)	**T9** (**°**)	**T10** (**°**)	**T11** (**°**)	**T12** (**°**)	**L1** (**°**)	**L2** (**°**)	**L3** (**°**)	**L4** (**°**)	**Pel** (**°**)	**Trunk** **Inclination** (**VP-DM**) (**°**)		**Trunk** **Inclination** (**VP-DM**) (**mm**)	**Thoracic** **Kyphosis** (**ICT-ITL**) (**°**)		**Lumbar** **Lordosis** (**ITL-ILS**) (**°**)
**AP vs. HM = 0** (**One-Sample** **Wilcoxon** **Signed** **Rank Test**)	**Observed** **Median**	-	25.01	25.24	23.95	19.04	13.65	10.36	7.91	4.83	0.23	−5.69	−10.98	−15.07	−17.42	−17.61	−14.08	−3.58	10.52	18.40	2.98		26.15	50.54		40.98
	**Standardized Test Statistic**	-	12.29	12.29	12.26	12.26	12.23	12.25	12.15	10.91	0.89	−11.74	−12.29	−12.26	−12.26	−12.26	−12.23	−4.97	11.74	12.27	11.84		11.84	12.26		12.29
	***p*-Value**	-	0.00 *	0.00 *	0.00 *	0.00 *	0.00 *	0.00 *	0.00 *	0.00 *	0.38	0.00 *	0.00 *	0.00 *	0.00 *	0.00 *	0.00 *	0.00 *	0.00 *	0.00 *	0.00 *		0.00 *	0.00 *		0.00 *
**Between-Subject Effects** (**two-way ANOVA**)	**Sex** (**Sig**)	-	0.05 *	0.80	0.00 *	0.00 *	0.00 *	0.00 *	0.00 *	0.00 *	0.00 *	0.00 *	0.00 *	0.00 *	0.00 *	0.32	0.01 *	0.00 *	0.00 *	0.10	0.74		0.68	0.66		0.00 *
	**Age Cohort** (**Sig**)	-	0.01 *	0.03 *	0.03 *	0.01 *	0.01 *	0.19	0.47	0.30	0.14	0.11	0.13	0.20	0.42	0.59	0.33	0.13	0.31	0.00 *	0.85		0.80	0.00 *		0.70
	Sex *Age **Cohort** (**Sig**)	-	0.84	0.50	0.35	0.44	0.77	0.93	0.70	0.67	0.70	0.62	0.49	0.24	0.26	0.95	0.84	0.26	0.12	0.86	0.24		0.22	0.60		0.58
	**Young vs.** **Old** (**Sig**)	-	0.38	0.07	0.01 *	0.00 *	0.00 *	0.15	1.00	0.84	0.30	0.16	0.13	0.20	0.56	0.80	0.99	1.00	0.94	0.00 *	0.96		0.96	0.00 *		0.52
	**Young vs. Middle** (**Sig**)	-	0.00 *	0.02 *	0.80	0.84	0.75	0.70	0.47	0.82	0.98	1.00	0.96	0.78	0.38	0.58	0.66	1.00	0.60	0.03 *	0.88		0.84	0.06		0.80
	**Middle vs. Old** (**Sig**)	-	0.21	0.84	0.06	0.01 *	0.06	1.00	1.00	0.34	0.21	0.20	0.22	0.71	0.99	0.98	0.46	0.22	0.40	0.34	0.99		0.99	0.02 *		0.89

**Bold** and * = *p* < 0.05. Abbreviations: Sh = shoulder; Pel = pelvis; AP = group of all participants; HM = hypothetical median; Sig = significance.

**Table 6:** Comparison of the results for the global spine parameters from the current study with those of previous research.

				Transversal Plane				Coronal Plane						Sagittal Plane			
				Surface Rotation RMS (°)	Surface Rotation MAX (°)	(Right Side) Surface Rotation +Max (°)	(Left Side) Surface Rotation −Max (°)	Trunk Imbalance (VP-DM) (°)	Trunk Imbalance (VP-DM) (mm)	Apical Deviation RMS (mm)	Apical Deviation MAX (mm)	(Right Side) Apical Deviation (VP-DM) +Max (mm)	(Left Side) Apical Deviation (VP-DM) −Max (mm)	Trunk Inclination (VP-DM) (°)	Trunk Inclination (VP-DM) (mm)	Thoracic Kyphosis (ICT-ITL) (°)	Lumbar Lordosis (ITL-ILS) (°)
**Current Study**	**All Participants**		MoM	2.3	−1.9	1.5	−3.3	−0.2	−1.7	3.6	−1.8	3.2	−4.9	3.1	26.0	49.9	40.9
			SD	0.9	4.0	1.4	2.3	0.8	7.3	1.7	7.1	3.0	3.3	2.1	17.5	8.3	9.2
	**Sex**	**All Females**	MoM	2.2	−1.8	1.5	−3.2	−0.2	−1.6	3.5	−2.3	2.9	−4.9	3.1	25.6	50.1	44.0
			SD	0.9	4.0	1.4	2.3	0.9	7.7	1.7	6.7	2.8	3.3	2.1	17.6	8.2	8.5
		**All Males**	MoM	2.3	−2.1	1.7	−3.6	−0.2	−2.0	3.8	−0.8	3.8	−4.7	3.0	26.7	49.6	34.9
			SD	0.9	4.0	1.4	2.3	0.8	6.6	1.6	7.7	3.4	3.2	2.0	17.4	8.6	7.3
	**Age Cohort** **“Young”**	**All Young** **Participants**	MoM	2.3	−2.3	1.4	−3.5	−0.3	−2.5	3.5	−0.9	3.3	−4.4	2.9	24.8	46.5	40.1
			SD	0.9	3.6	1.2	2.2	0.8	6.5	1.6	7.0	2.9	3.1	2.2	18.4	7.1	8.9
		**Young** **Females**	MoM	2.4	−2.4	1.3	−3.5	−0.3	−2.6	3.4	−1.1	3.1	−4.4	2.8	22.6	46.9	43.0
			SD	0.9	3.8	1.3	2.3	0.8	6.7	1.6	7.2	2.7	3.1	2.1	17.5	6.9	8.7
		**Young** **Males**	MoM	2.2	−2.3	1.7	−3.6	−0.2	−2.3	3.6	−0.4	3.8	−4.4	3.3	28.9	45.8	34.5
			SD	0.8	3.4	1.2	2.2	0.7	6.3	1.6	6.7	3.1	3.1	2.2	19.7	7.7	6.5
	**Age Cohort** **“Middle”**	**All** **Middle** **Participants**	MoM	2.1	−2.3	1.4	−3.4	−0.2	−1.9	3.8	−2.9	2.9	−5.3	3.2	27.0	49.7	41.0
			SD	1.0	3.6	1.4	2.2	0.8	7.0	1.8	7.0	3.2	3.3	2.0	17.0	7.7	9.0
		**Middle** **Females**	MoM	2.0	−2.2	1.3	−3.3	−0.2	−1.8	3.6	−3.3	2.8	−5.3	3.2	26.3	49.3	43.7
			SD	0.8	3.4	1.4	2.0	0.9	7.4	1.9	6.4	2.9	3.3	2.1	17.3	7.6	9.0
		**Middle** **Males**	MoM	2.3	−2.4	1.5	−3.6	−0.2	−2.2	4.1	−2.2	3.2	−5.4	3.2	28.3	50.5	35.7
			SD	1.1	3.8	1.4	2.6	0.7	6.2	1.7	8.0	3.7	3.4	1.9	16.7	7.9	6.2
	**Age Cohort** **“Old”**	**All Old** **Participants**	MoM	2.3	−1.1	1.8	−3.1	−0.1	−0.8	3.6	−1.5	3.3	−4.8	3.1	26.1	53.4	41.6
			SD	0.9	4.6	1.7	2.4	0.9	8.3	1.6	7.3	3.1	3.3	2.1	17.4	8.7	9.7
		**Old** **Females**	MoM	2.3	−0.9	1.8	−2.9	−0.2	−0.4	3.5	−2.4	2.8	−5.1	3.4	27.8	54.0	45.3
			SD	0.9	4.6	1.7	2.4	1.0	8.8	1.7	6.5	2.7	3.5	2.2	18.2	8.7	7.7
		**Old** **Males**	MoM	2.5	−1.4	1.8	−3.5	−0.1	−1.4	3.7	0.3	4.4	−4.3	2.6	22.8	52.4	34.4
			SD	0.9	4.8	1.7	2.3	0.9	7.4	1.6	8.5	3.6	3.0	1.8	15.5	9.0	9.1
**Literature Comparison**	**Degenhardt et al., 2017 [22]**	**Young-Middle Participants**	Mean	3.8	1.8	5.6	−4.6	0.1	1.0	5.6	3.6 ‡	7.9	−5.0	3.1	26.0	48.1	35.6
			SD	1.4	7.2	3.4	2.9	0.8	7.2	3.0	10.3 ‡	5.8	4.1	2.3	18.7	9.1	8.4
	**Degenhardt et al., 2020 [23]**	**Young-Middle Participants**	Mean	3.8	2.0	5.7	−4.5	0.2	1.3	5.4	4.3	8.0	−4.6	3.2	26.2	48.5	35.4
			SD	1.0	6.0	2.8	2.4	0.7	5.6	2.5	8.7	5.1	2.9	2.2	17.7	8.3	7.6
	**Michalik et al., 2020 [19]**	**Young** **Females**	Mean	3.6	-	-	-	−0.1	-	5.6	-	-	-	2.1	-	44.0 †	37.4 †
			SD	1.6	-	-	-	0.9	-	2.3	-	-	-	2.4	-	8.6 †	9.8 †
		**Young** **Males**	Mean	3.5	-	-	-	−0.1	-	5.1	-	-	-	1.9	-	44.6 †	29.0 †
			SD	1.6	-	-	-	1.0	-	2.1	-	-	-	1.9	-	7.8 †	7.7 †
	**Schröder et al., 2011 [20]**	**Young** **Females**	Mean	3.6	-	-	-	-	6.9	5.5	-	-	-	-	12.3	47.1	42.7
			SD	1.8	-	-	-	-	4.6	2.3	-	-	-	-	17.9	8.6	8.2
		**Young** **Males**	Mean	3.1	-	-	-	-	7.7	5.8	-	-	-	-	10.3	49.2	35.8
			SD	1.5	-	-	-	-	7.2	2.5	-	-	-	-	16.4	9.3	6.6
	**Schröder et al., 2014 [21]**	**Young** **Females**	Mean	3.4	-	-	-	-	7.7	4.2	-	-	-	16.6 *	-	45.4	44.0
			SD	1.7	-	-	-	-	4.6	2.0	-	-	-	15.5 *	-	8.1	9.4
		**Young** **Males**	Mean	3.6	-	-	-	-	6.9	4.5	-	-	-	20.8 *	-	47.2	35.9
			SD	1.4	-	-	-	-	4.6	2.1	-	-	-	15.2 *	-	7.3	8.2
	**Furian et al., 2013 [18]**	**Girls**	Mean	-	-	-	-	-	5.7	-	4.9 ‡	-	-	-	2.6 *	47.1 †∆	42.1 †∆
			SD	-	-	-	-	-	0.7	-	0.7 ‡	-	-	-	0.7 *		
		**Boys**	Mean	-	-	-	-	-	7.4	-	4.7 ‡	-	-	-	3.0 *	7.5 †∆	9.9 †∆
			SD	-	-	-	-	-	0.8	-	0.4 ‡	-	-	-	0.2 *		

Abbreviations: MoM = mean of means; SD = standard deviation; ‡ = parameters could not be clearly assigned; * = values not comprehensible (unit of measurement questionable); † = parameters differ slightly from those used in the current study (“Thoracic Kyphosis VP-T12”; “Lumbar Lordosis T12-DM”); ∆ = results are only available as one mean and SD for the entire group (*n* = 345) and not for the subgroups, respectively. [22]: *n* = 30 participants, age = 30.2 ± 9.8 years; [23]: *n* = 29 participants, age = 30.1 ± 10.1 years; [19]: *n* = 56 females, age = 23.6 ± 2.0 years and *n* = 65 males, age = 24.3 ± 2.2 years; [20]: *n* = 89 females, age = 26.4 ± 4.5 years and *n* = 88 males, age = 27.7 ± 4.4 years; [21]: *n* = 52 females, age = 26.1 ± 6.9 years and *n* = 51 males, age = 28.2 ± 7.4 years; [18]: *n* = 168 girls, age = 8.3 ± 1.3 years and *n* = 177 boys, age = 8.6 ± 1.2 years.

## Data Availability

Due to ethical and privacy reasons, the data presented in this study are not publicly available.
